# Identification and analysis of genes associated with epithelial ovarian cancer by integrated bioinformatics methods

**DOI:** 10.1371/journal.pone.0253136

**Published:** 2021-06-18

**Authors:** Ting Gui, Chenhe Yao, Binghan Jia, Keng Shen

**Affiliations:** 1 Department of Obstetrics and Gynecology, Peking Union Medical College Hospital, Peking Union Medical College, Chinese Academy of Medical Sciences, Beijing, China; 2 Department of R&D Technology Center, Beijing Zhicheng Biomedical Technology Co, Ltd, Beijing, China; University of Calgary, CANADA

## Abstract

**Background:**

Though considerable efforts have been made to improve the treatment of epithelial ovarian cancer (EOC), the prognosis of patients has remained poor. Identifying differentially expressed genes (DEGs) involved in EOC progression and exploiting them as novel biomarkers or therapeutic targets is of great value.

**Methods:**

Overlapping DEGs were screened out from three independent gene expression omnibus (GEO) datasets and were subjected to Gene ontology (GO) and Kyoto encyclopedia of genes and genomes (KEGG) pathway enrichment analyses. The protein-protein interactions (PPI) network of DEGs was constructed based on the STRING database. The expression of hub genes was validated in GEPIA and GEO. The relationship of hub genes expression with tumor stage and overall survival and progression-free survival of EOC patients was investigated using the cancer genome atlas data.

**Results:**

A total of 306 DEGs were identified, including 265 up-regulated and 41 down-regulated. Through PPI network analysis, the top 20 genes were screened out, among which 4 hub genes, which were not researched in depth so far, were selected after literature retrieval, including CDC45, CDCA5, KIF4A, ESPL1. The four genes were up-regulated in EOC tissues compared with normal tissues, but their expression decreased gradually with the continuous progression of EOC. Survival curves illustrated that patients with a lower level of CDCA5 and ESPL1 had better overall survival and progression-free survival statistically.

**Conclusion:**

Two hub genes, CDCA5 and ESPL1, identified as probably playing tumor-promotive roles, have great potential to be utilized as novel therapeutic targets for EOC treatment.

## Introduction

Ovarian cancer has the highest mortality in gynecologic cancers and most patients are diagnosed at advanced stages [1]. Many patients would still relapse even if they are treated with satisfied cytoreductive surgery (CRS) combined with standard platinum-based chemotherapy. The 5-year survival rate for patients with advanced ovarian cancer is about 30% [2]. Thus, investigating effective molecular markers and understanding essential genes involved in the biological process of ovarian cancer is in urgent necessity.

Gene expression profiling is a powerful strategy, based on which differentially expressed genes (DEGs) could be screened out between patients and healthy population [3]. DEGs could be used to explore the molecular signal pathways and to analyze the gene regulatory networks in various diseases including epithelial ovarian cancer (EOC). At present, thousands of DEGs have been found that might be involved in the development and progression of ovarian cancer [4], but the results are inconsistent due to tissue heterogeneity, sample size, and different bioinformatics analyses methods and detection platforms. The analysis of individual experiments has high risk of bias, and integrated analyses of multiple databases could improve the representativity and reliability of the discovery of DEGs.

Microarray, as a large-scale technique for uncovering genetic alterations, has been widely used for collecting gene expression profiling data [5,6]. With the widespread application of microarray technology, substantial data have been published on public database platforms, among which the Gene Expression Omnibus (GEO) database is an important one [7]. Several studies have been reported using bioinformatics analysis to identify DEGs in EOC based on GEO database. For example, Yang et al. identified 17 most closely related DEGs from the protein-protein interaction network involved in EOC [8]. Feng et al. identified four significant up-regulated DEGs (BUB1B, BUB1, TTK and CCNB1) with poor prognosis in EOC patients [9]. Besides, Lou et al. reported three genes (GJB2, S100A2, SPOCK2) significantly up-regulated in advance stage than in early stage of ovarian cancer, and elucidated a regulatory role of pseudogene /lncRNA-hsa-miR-363-3p-SPOCK2 pathway in progression of EOC [10].

In our study, three microarray datasets were downloaded from the GEO database, and analyses were conducted to obtain overlapping DEGs in EOC. Then, functional enrichment and network construction were implemented to evaluate the underlying molecular mechanisms possibly involved in carcinogenesis and tumor progression. Finally, the hub genes potentially playing essential roles in EOC were identified and validated, and their correlation with EOC patient survival were explored. We hope our integrative analyses could be in help of providing more reliable targets for exploration of the molecular mechanisms as well as effective treatment in EOC.

## Materials and methods

### Data acquisition and DEGs identification

“Epithelial ovarian cancer” was searched in GEO database (www.ncbi.nlm.nih.gov/geo), with the inclusion criteria of human species and inclusion of mRNA in the microarray data. Three microarry expression profile datasets, GSE119056, GSE54388, GSE66957, were downloaded.

GEO2R, an interactive web tool (http://www.ncbi.nlm.nih.gov/geo/geo2r) comparing two groups of samples under the same conditions, was used to find DEGs between EOC and adjacent normal ovarian tissues. p < 0.05 and |log FC |>1 were set as the threshold.

### Functional annotation and Pathway enrichment

The overlapping DEGs from three GEO datasets were subjected to Gene ontology **(**GO) and Kyoto encyclopedia of genes and genomes **(**KEGG) pathway analysis [11]. p < 0.05 was considered as having statistical significance.

### Protein-protein interaction (PPI) network construction

Protein-protein interaction (PPI) network was constructed by Cytoscape software (http://www.cytoscape.org), based on the Search Tool for the Retrieval of Interacting Genes (STRING) database (http://string-db.org) [12,13]. Degree > 10 was set as cut-off threshold. The top 20 genes were selected as hub genes.

### Hub genes validation by two databases

The relative expression of selected hub genes was validated by two databases, Gene Expression Omnibus (GEO) and Gene Expression Profiling Interactive Analysis (GEPIA). GEO, an online platform providing microarray datasets (http://www.ncbi.nlm.nih.gov/geo/), is widely used to validate the expression of specific genes facilitating the finding of potential key genes involved in carcinogenesis and tumor progression [14]. GEPIA, an online tool providing data concerning gene expression, tumor stage/grade, and survival (http://gepia.cancer-pku.cn/), is widely adopted to compare the gene expression between tumor and normal tissues, based on the Cancer Genome Atlas (TCGA) and the Genotype-Tissue Expression (GTEx) [15]. In our study, relative expression of hub genes was detected with a fold change of 2 and a threshold of p < 0.05.

### Tumor stage/grade and survival analysis

UCSC Xena, a free online database (http://xenabrowser.net/), was utilized to analyze the contribution of hub genes to tumor stage/grade and overall survival (OS) and progression-free survival (PFS) from TCGA samples. Patients were classified into two groups, a relatively high expression group and a relatively low expression group. p < 0.05 was considered as having statistical significance.

## Results

### 1. Identification of DEGs

**Fig 1:**
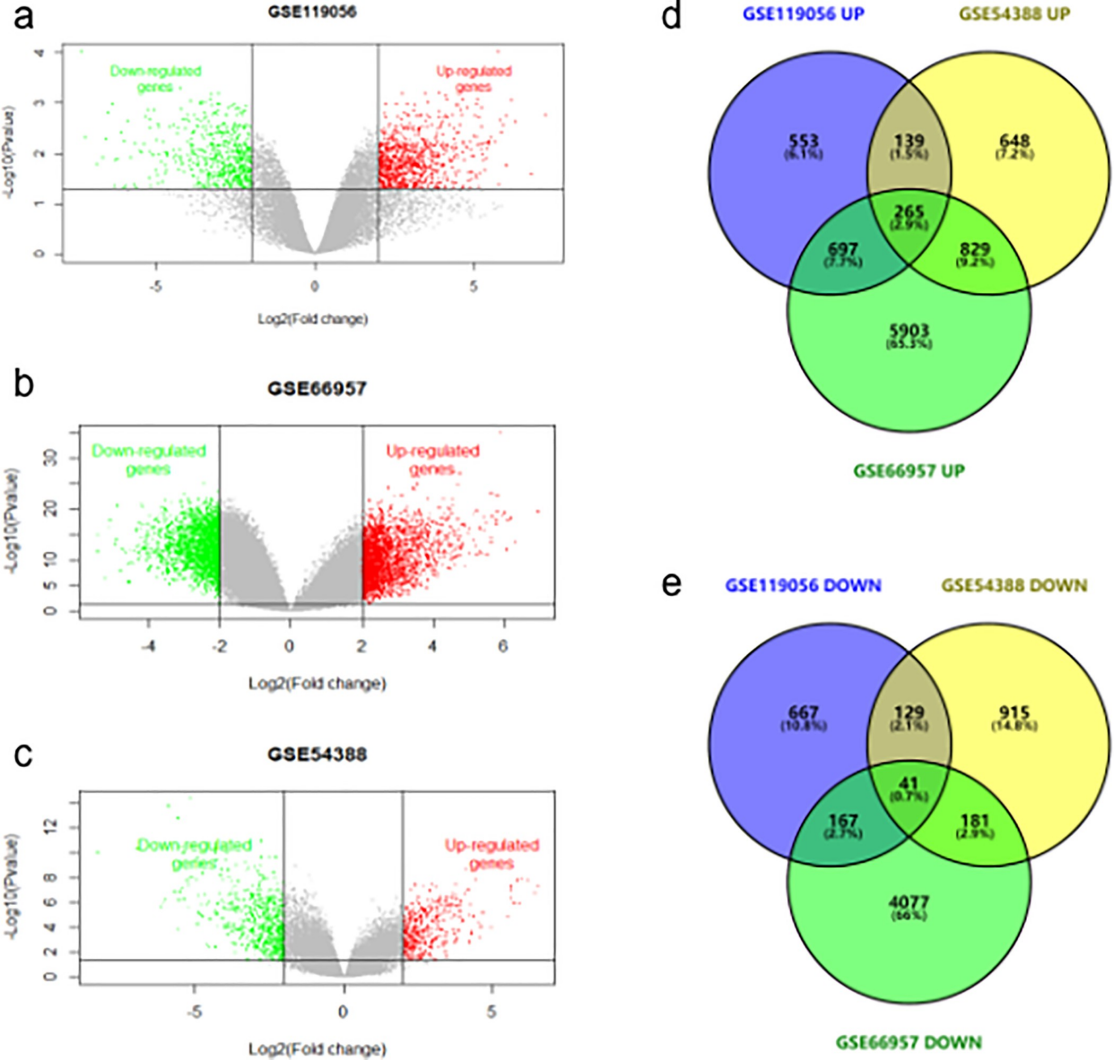
Identification of differentially expressed genes (DEGs) in three gene expression omnibus (GEO) datasets. (a) Volcano plot of DEGs in GSE119056. (b) Volcano plot of DEGs in GSE54388. (c) Volcano plot of DEGs in GSE66957. Red, blue and gray color represent relatively high, low and equal expression of genes in the corresponding group, respectively. (d) Venn diagram of 265 overlapping up-regulated genes from intersection of three independent GEO datasets. p < 0.05 and |log FC |>1 were set as the threshold. (e) Venn diagram of 41 overlapping down-regulated genes from intersection of three independent GEO datasets. p < 0.05 and |log FC |>2 were set as the threshold.

The volcano plot of each gene expression profile data was shown in Fig 1A–1C. Red or blue dots represent significantly up-regulated or down-regulated genes, respectively. A total of 306 overlapping DEGs, including 265 up-regulated and 41 down-regulated, were identified from the intersection of three microarray datasets by comparing EOC tissues with adjacent normal ovarian tissues under the criteria (Fig 1D and 1E).

### 2. Functional analyses of the overlapping DEGs

**Fig 2:**
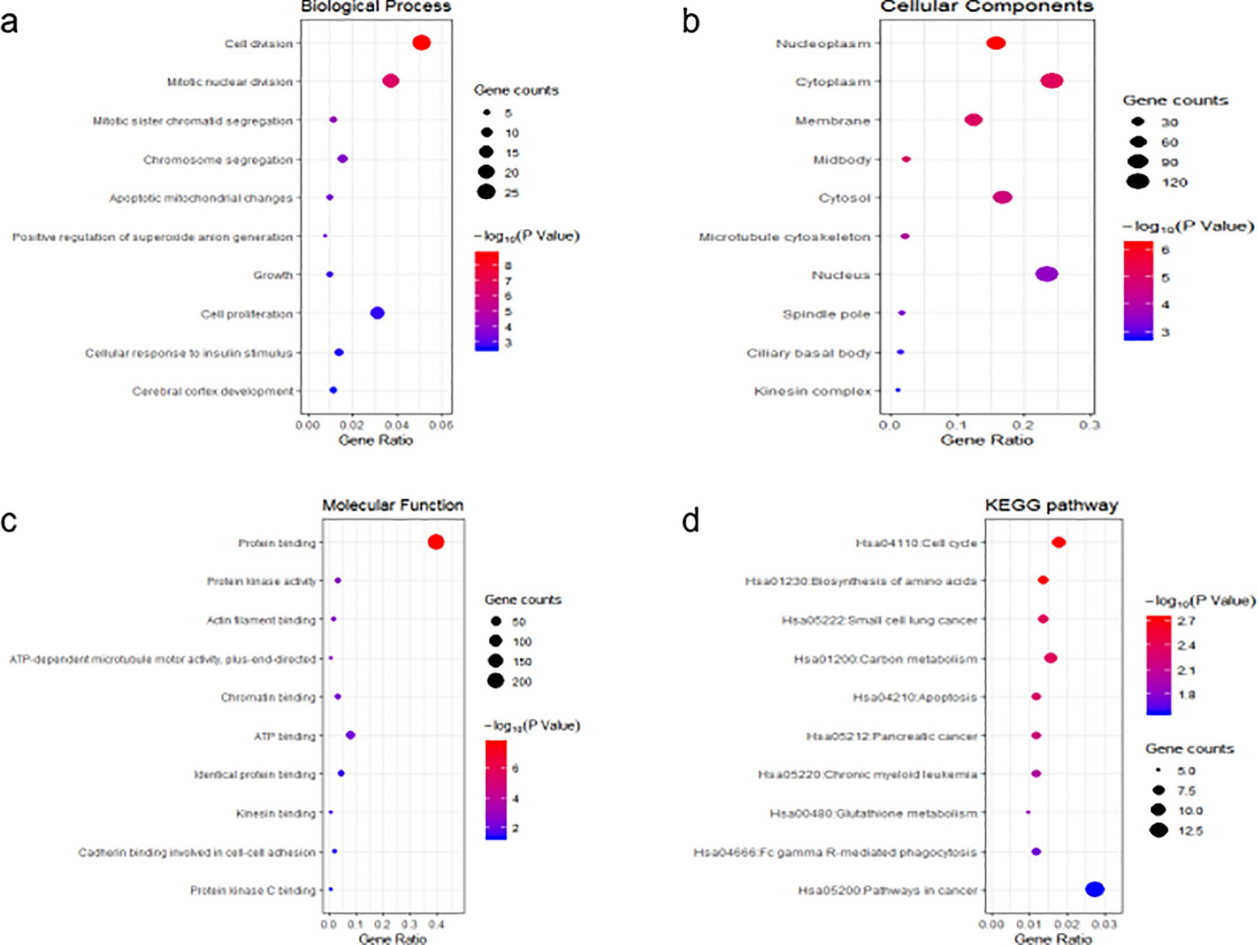
GO analysis and KEGG pathway analysis of the overlapping DEGs. (a) Top 10 of biological process. (b) Top 10 of cellular component. (c) Top 10 of molecular function. (d) Top 10 of KEGG pathway enrichment.

GO analysis and KEGG pathway enrichment analysis were performed for the overlapping DEGs. The top 10 biological processes that these DEGs involved in was presented in Fig 2A, among which cell division, cell proliferation, adhesion, and response to drug were closely associated with cancer progression. Concerning cellular component, GO analysis results showed that the overlapping DEGs were mainly enriched in cytoplasm, nucleus, cell membrane, and extracellular exosome (Fig 2B). Regarding molecular function classification, the DEGs were significantly enriched in the following functions: protein binding, ATP binding, poly A RNA binding, and chromatin binding (Fig 2C). The results from KEGG analysis showed that these DEGs were particularly enriched in pathways in cancer, cell cycle, and carbon metabolism (Fig 2D).

### 3. PPI netwok construction and hub genes identification

**Fig 3:**
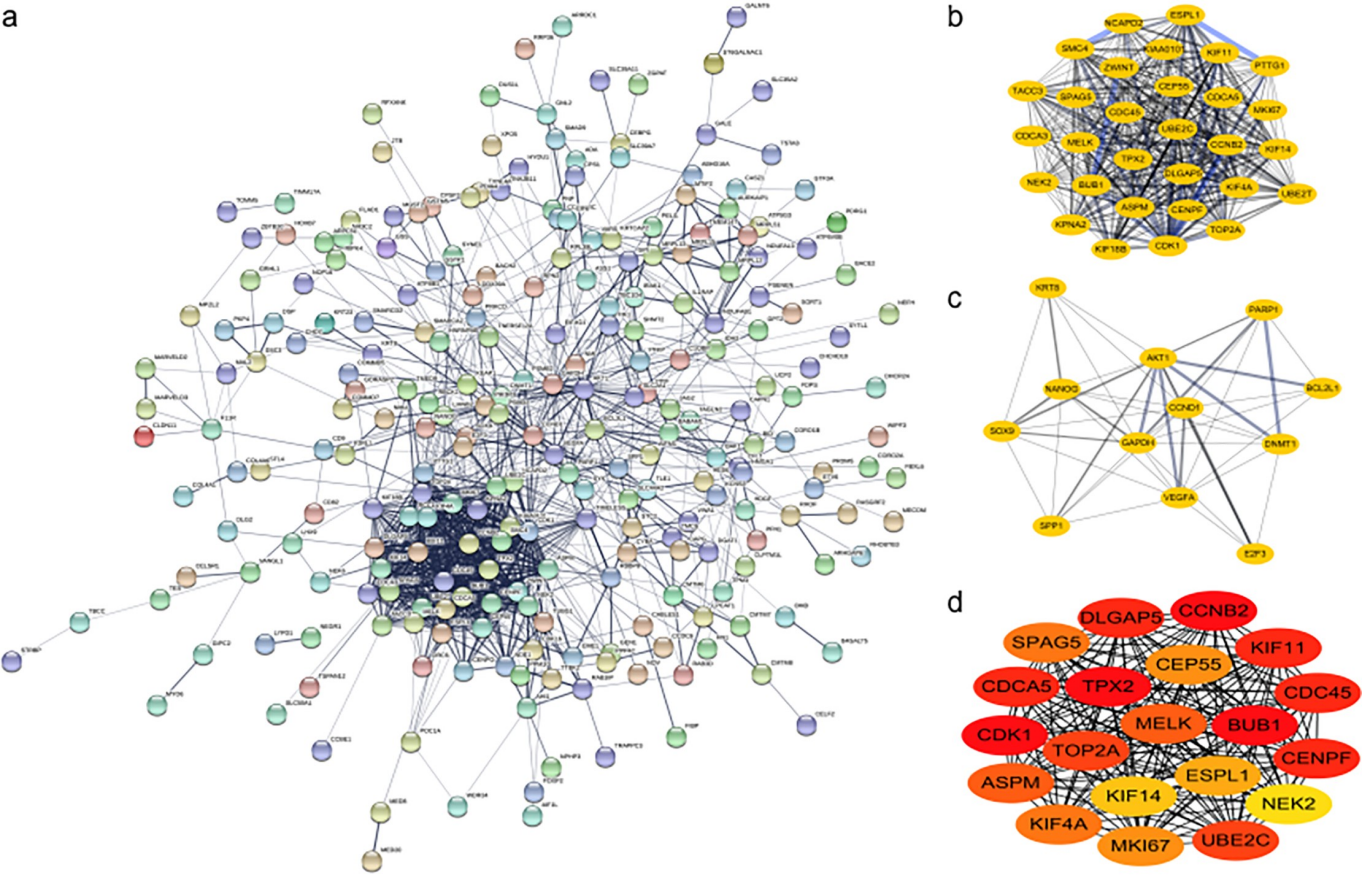
Protein-protein interactions (PPI) network, module analysis, and hub gene identification. (a) PPI network of overlapping differentially expressed genes was constructed in STRING database. (b-c) Top two modules screened using Molecular Complex Detection (MCODE) in Cytoscape software. (d) Top twenty hub genes selected by the CytoHubba in Cytoscape based on the degree of each protein node.

The PPI network of DEGs was constructed (Fig 3A), and the top two modules were identified (Fig 3B and 3C). Overall, the number of nodes was 306, the number of edges was 1105, the average node degree was 7.22, the average local clustering coefficient was 0.424, and the PPI enrichment p-value was < 1.0e-16. For details, module 1 consisted of 30 nodes and 416 edges. For module 2, 12 nodes and 46 edges existed. Finally, the top 20 genes were selected by using the MCC method (Fig 3D, Table 1), including BUB1, CDK1, CCNB2, TPX2, KIF11, CDC45, CENPF, DLGAP5, CDCA5, UBE2C, TOP2A, ASPM, MELK, KIF4A, SPAG5, MKI67, CEP55, ESPL1, KIF14, and NEK2.

**Table 1:** Top 20 hub genes in network ranked by MCC method.

Rank	Name	Score
1	BUB1	3.62E+22
1	CDK1	3.62E+22
1	CCNB2	3.62E+22
1	TPX2	3.62E+22
5	KIF11	3.62E+22
5	CDC45	3.62E+22
5	CENPF	3.62E+22
5	DLGAP5	3.62E+22
5	CDCA5	3.62E+22
10	UBE2C	3.62E+22
10	TOP2A	3.62E+22
12	ASPM	3.62E+22
12	MELK	3.62E+22
14	KIF4A	3.62E+22
14	SPAG5	3.62E+22
16	MKI67	3.62E+22
16	CEP55	3.62E+22
18	ESPL1	3.39E+22
19	KIF14	3.39E+22
20	NEK2	3.38E+22

### 4. Literature retrieval of hub genes in Pubmed

Literature retrieval of the above 20 hub genes through PubMed, the professional literature retrieval database, showed that 16 genes had already been proven to play a role in EOC by multiple studies, while the left 4 genes had only 1 or 2 research papers published in until now, including CDC45, CDCA5, KIF4A, and ESPL1. Although there was only 1 research paper found focusing on the gene SPAG5, the correlation of SPAG5 with the ovarian cancer had been explored deeply in the paper. Thus, we selected the other four genes, CDC45, CDCA5, ESPL1, and KIF4A, as the subsequent hub genes in our subsequent research. Specific details of literature retrieval were summarized in Table 2.

**Table 2:** Literature retrieval of the top 20 Hub genes in three GSE datasets.

Name	No. of article	GSE119056			GSE54388			GSE66957		
		adj.P.value	P.value	log2FC	adj.P.value	P.value	log2FC	adj.P.value	P.value	log2FC
BUB1	25	1.03E-02	2.27E-04	3.7643571	4.61E-07	1.27E-09	3.23855602	7.05E-03	4.49E-03	1.5398069
CDK1	77	4.21E-02	3.52E-03	2.3614095	1.48E-06	7.18E-09	4.24011987	1.07E-02	7.05E-03	1.1646547
CCNB2	15	3.17E-02	2.16E-03	3.1905788	2.23E-06	1.22E-08	3.07033063	5.29E-04	2.82E-04	2.0113827
TPX2	11	6.39E-03	7.47E-05	5.4661937	2.44E-05	2.75E-07	2.6496947	1.89E-04	9.40E-05	2.1604674
KIF11	15	2.10E-02	1.03E-03	2.694089	3.63E-07	8.89E-10	3.42819271	1.58E-02	1.06E-02	1.148118
**CDC45**	**1**	1.16E-02	2.94E-04	3.7095745	1.83E-03	8.64E-05	1.39231177	5.31E-03	3.31E-03	1.3575417
CENPF	4	3.15E-02	2.14E-03	2.5105527	5.35E-06	3.86E-08	2.86744827	1.86E-03	1.08E-03	1.4950478
DLGAP5	6	2.69E-02	1.62E-03	3.0988429	4.45E-07	1.20E-09	3.65472057	3.77E-02	2.71E-02	1.247308
**CDCA5**	**1**	2.25E-02	1.17E-03	4.5519674	1.30E-04	2.56E-06	1.735301	8.13E-05	3.82E-05	2.0720946
UBE2C	10	4.79E-02	4.44E-03	2.5794941	2.83E-04	7.20E-06	2.27206271	1.57E-04	7.70E-05	2.1154632
TOP2A	149	3.05E-03	1.66E-05	5.2386187	4.77E-06	3.37E-08	3.90640641	3.15E-03	1.89E-03	1.764505
ASPM	6	1.67E-02	6.37E-04	4.057857	4.77E-04	1.43E-05	1.34713648	2.39E-02	1.66E-02	1.2680474
MELK	5	3.12E-02	2.11E-03	3.6767911	4.83E-07	1.38E-09	3.4296668	1.74E-02	1.18E-02	1.4320461
**KIF4A**	**1**	6.50E-03	7.90E-05	1.9508565	3.85E-06	2.56E-08	2.73378427	1.50E-03	8.55E-04	1.658541
SPAG5	1	2.53E-02	1.45E-03	2.3906094	3.59E-03	2.17E-04	1.28699292	4.58E-04	2.41E-04	1.5868884
MKI67	467	1.82E-03	5.02E-06	6.025098	4.89E-05	6.76E-07	1.75615999	9.37E-04	5.18E-04	1.7415113
CEP55	4	5.76E-03	5.84E-05	5.4686269	8.89E-08	1.22E-10	4.1695877	2.88E-02	2.03E-02	1.242852
**ESPL1**	**2**	3.53E-02	2.60E-03	1.6146454	4.17E-04	1.21E-05	1.46977455	7.22E-05	3.36E-05	1.5732776
KIF14	8	1.13E-02	2.78E-04	4.996634	1.84E-07	3.33E-10	3.67013213	3.17E-02	2.25E-02	1.1901737
NEK2	7	8.36E-03	1.40E-04	5.2428192	2.17E-09	1.07E-12	4.11000516	2.79E-03	1.66E-03	1.5497053

### 5. Validation of hub genes expression

**Fig 4:**
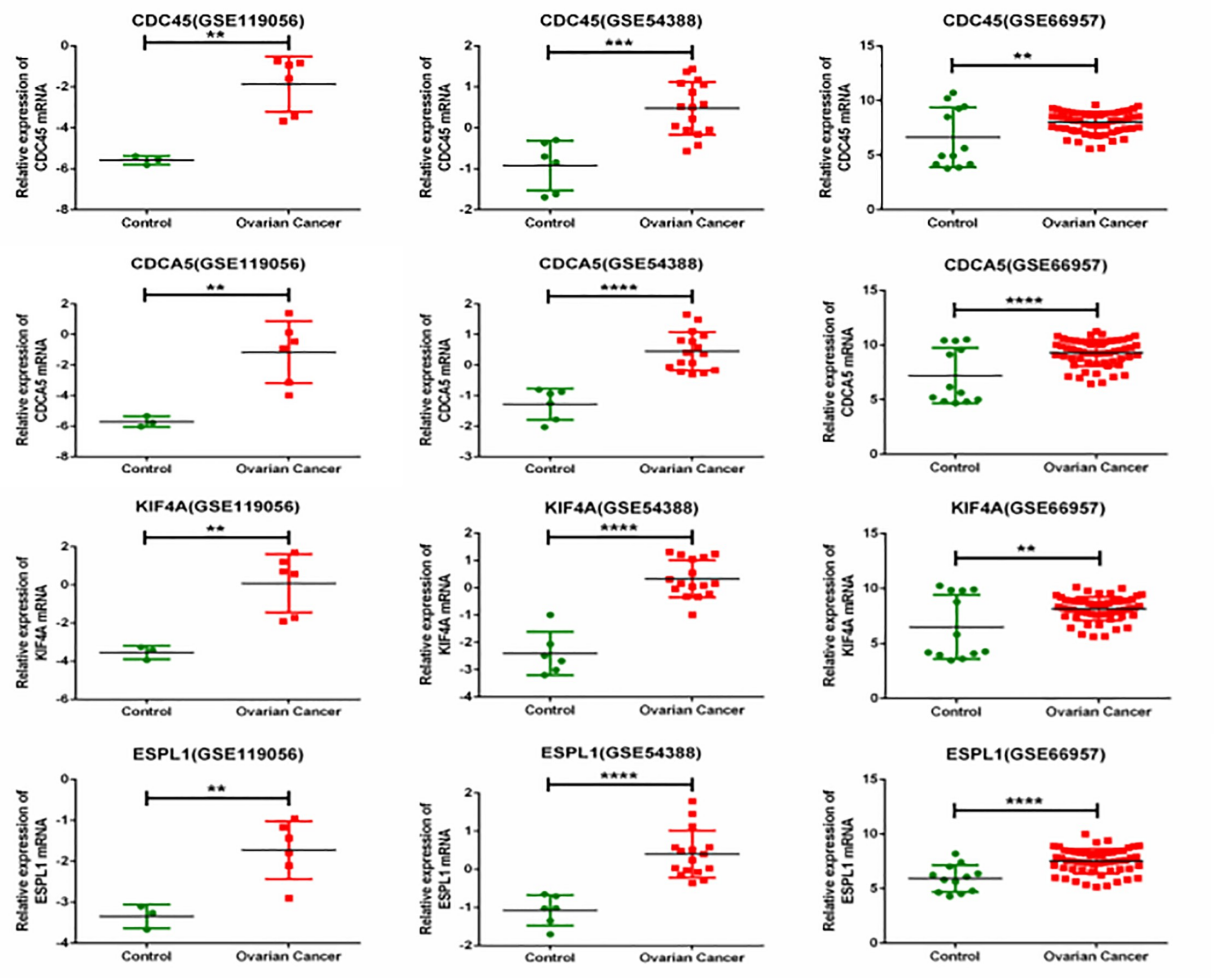
Validation of hub gene expression levels in GEO. The expression of CDC45, CDCA5, KIF4A, and ESPL1 in EOC tissues were significantly elevated compared with adjacent ovarian tissues. (a) CDC45; (b) CDCA5; (c) KIF4A; (d) ESPL1; *p < 0.05.

**Fig 5:**
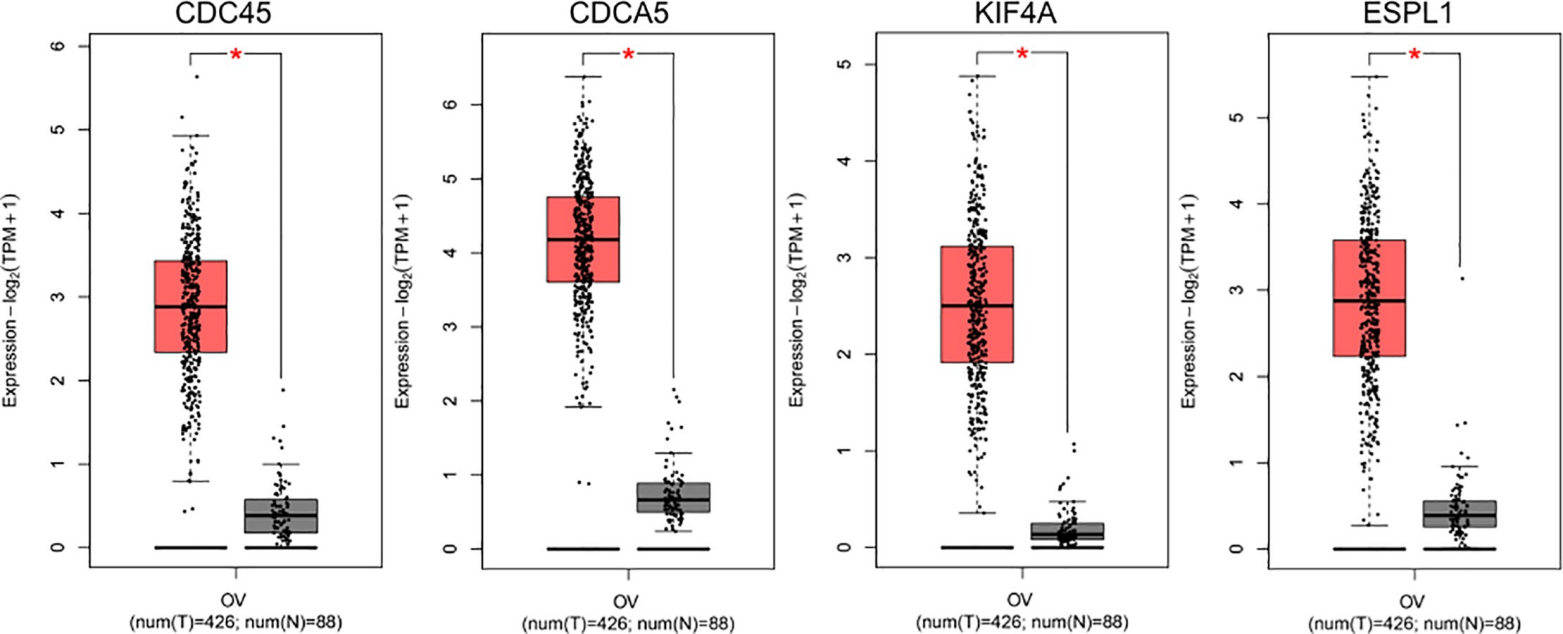
Validation of hub gene expression levels in GEPIA. The expression of CDC45, CDCA5, KIF4A, and ESPL1 in EOC tissues were significantly elevated compared with adjacent ovarian tissues. (a) CDC45; (b) CDCA5; (c) KIF4A; (d) ESPL1; *p < 0.05.

Transcriptional expression differences of hub genes were determined between EOC tissues and adjacent normal ovarian tissues in GEO and GEPIA. As shown in Fig 4 (GEO) and Fig 5 (GEPIA), mRNA levels of four hub genes, CDC45, CDCA5, ESPL1, and KIF4A, were all significantly up-regulated in EOC samples compared with normal ovarian tissues.

### 6. Clinical stage analyses of hub genes

**Fig 6:**
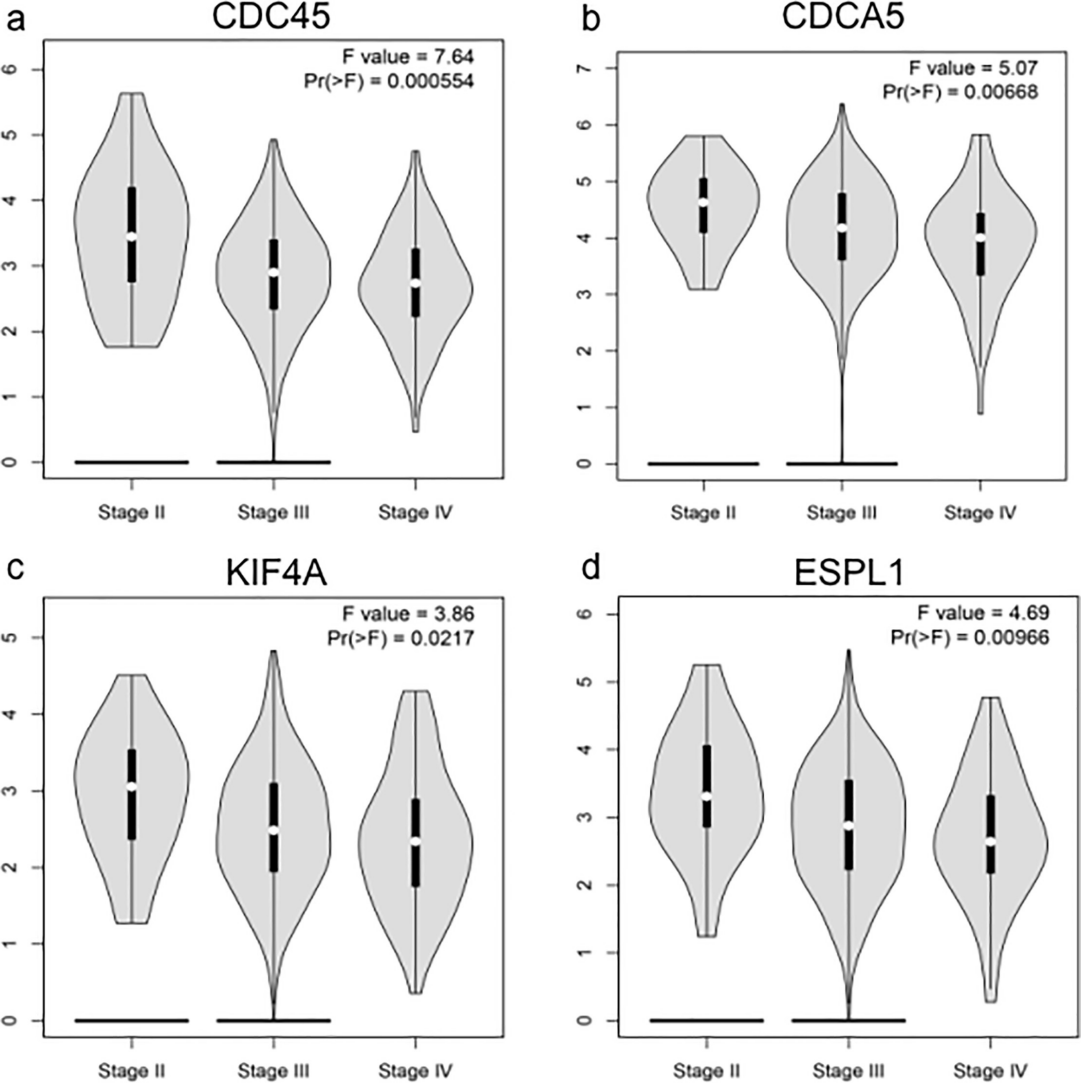
The expression level of hub genes in EOC tissues at different stages. To further verify the expression level of the hub genes in EOC tissues at different stages, the hub genes were analyzed by the GEPIA2 online database. ANOVA was performed to assess the statistical significance of the variations. Pr (>F) < 0.05 was considered statistically significant. According to the result, there were significant variations in the expression levels of CDC45, CDCA5, KIF4A, and ESPL1 (a-d). The overall trends indicated that the expression of these four genes decreased gradually with the continuous progression of OC.

The expression levels of the 4 hub genes at different tumor stages were shown in Fig 6. According to the result, it was easy to find that there were significant variations in the expression levels of CDC45 [Pr(>F) = 0.000554], CDCA5 [Pr(>F) = 0.00668], KIF4A [Pr(>F) = 0.0217], and ESPL1 [Pr(>F) = 0.00966]. The overall trends indicated that the expression of these four genes decreased gradually with the continuous progression of EOC, although the overall expression in EOC tissues were significantly higher than that in normal ovarian tissues as mentioned above.

### 7. Survival analyses of hub genes

**Fig 7:**
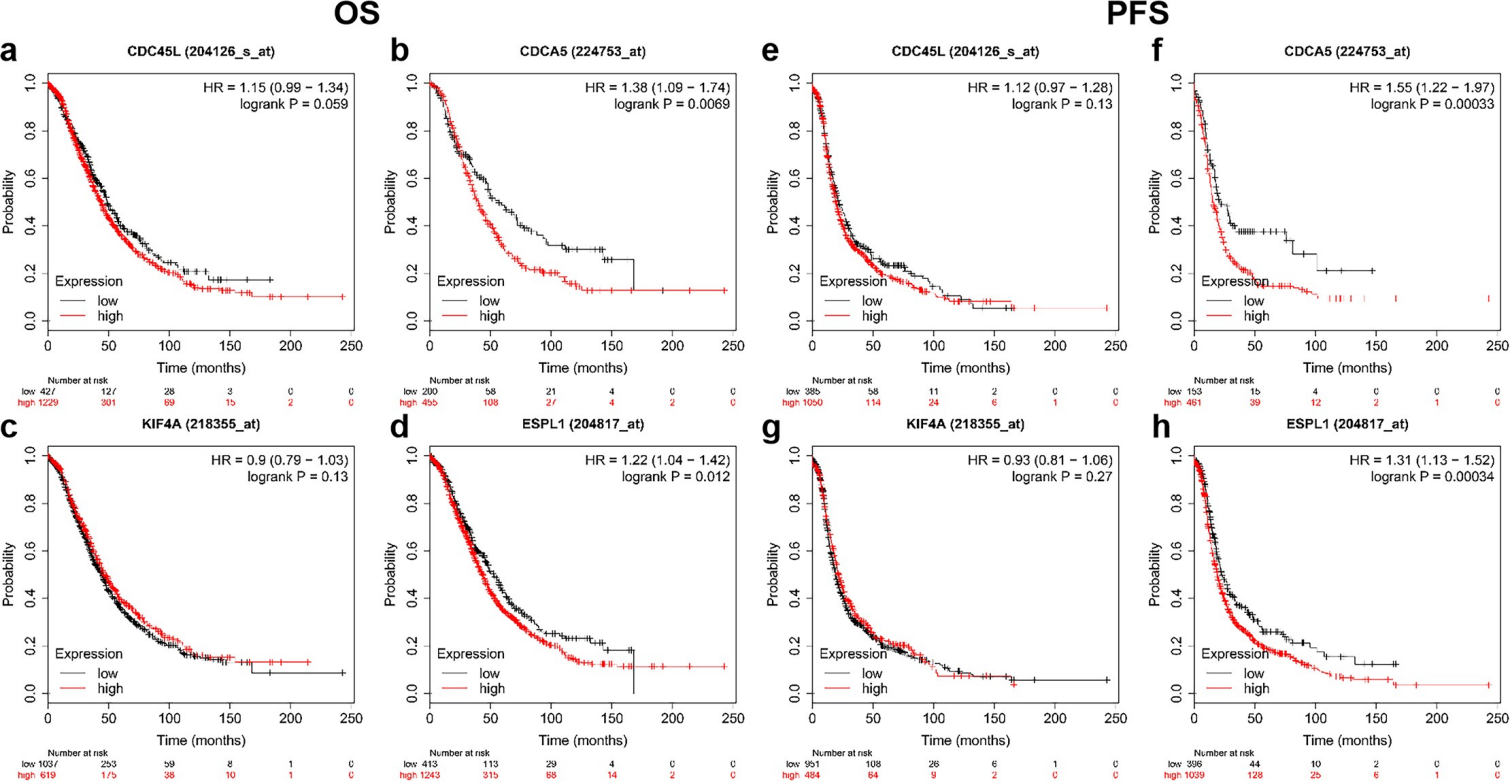
Overall survival and progression-free survival curves of DEGs in patients with EOCs from TCGA database. The relatively higher expression of CDC45 had no statistical influence on patients’ overall survival. (b) The relatively higher expression of CDCA5 was associated with poor prognosis of EOC patients. (c) The relatively higher expression of KIF4A had no statistical influence on patients’ overall survival. (d) The relatively higher expression of ESPL1 was associated with poor prognosis of EOC patients. (e) The relatively higher expression of CDC45 had no statistical influence on patients’ progression-free survival. (f) The relatively higher expression of CDCA5 was associated with poor prognosis of EOC patients. (g) The relatively higher expression of KIF4A had no statistical influence on patients’ progression-free survival. (h) The relatively higher expression of ESPL1 was associated with poor prognosis of EOC patients.

To further investigate the prognostic values of hub genes in EOC, we conducted survival analyses based on the TCGA data downloaded from the UCSC Xena database. As suggested in Fig 7A–7D, the relatively higher expression of CDCA5 and ESPL1 was associated with poor prognosis of EOC patients, coherent with higher expression in EOC tissues vs. lower expression in normal ovarian tissues, while the other two genes, CDC45 and KIF4A, had no statistical influence on patients’ overall survival.

Furthermore, we also detected whether these genes were related to the progression-free survival, and survival curves illustrated that CDCA5 and ESPL1 notably affected the progression-free survival time of EOC patients (Fig 7E–7H). Evidently, patients with a lower level of CDCA5 and ESPL1 had better progression-free survival compared to patients with higher CDCA5 and ESPL1 expression.

From the analysis above, we concluded that CDCA5 and ESPL1 might be closely correlated with EOC overall and progression-free survival, implying the essential roles that these two genes might play in EOC progression.

**Fig 8:**
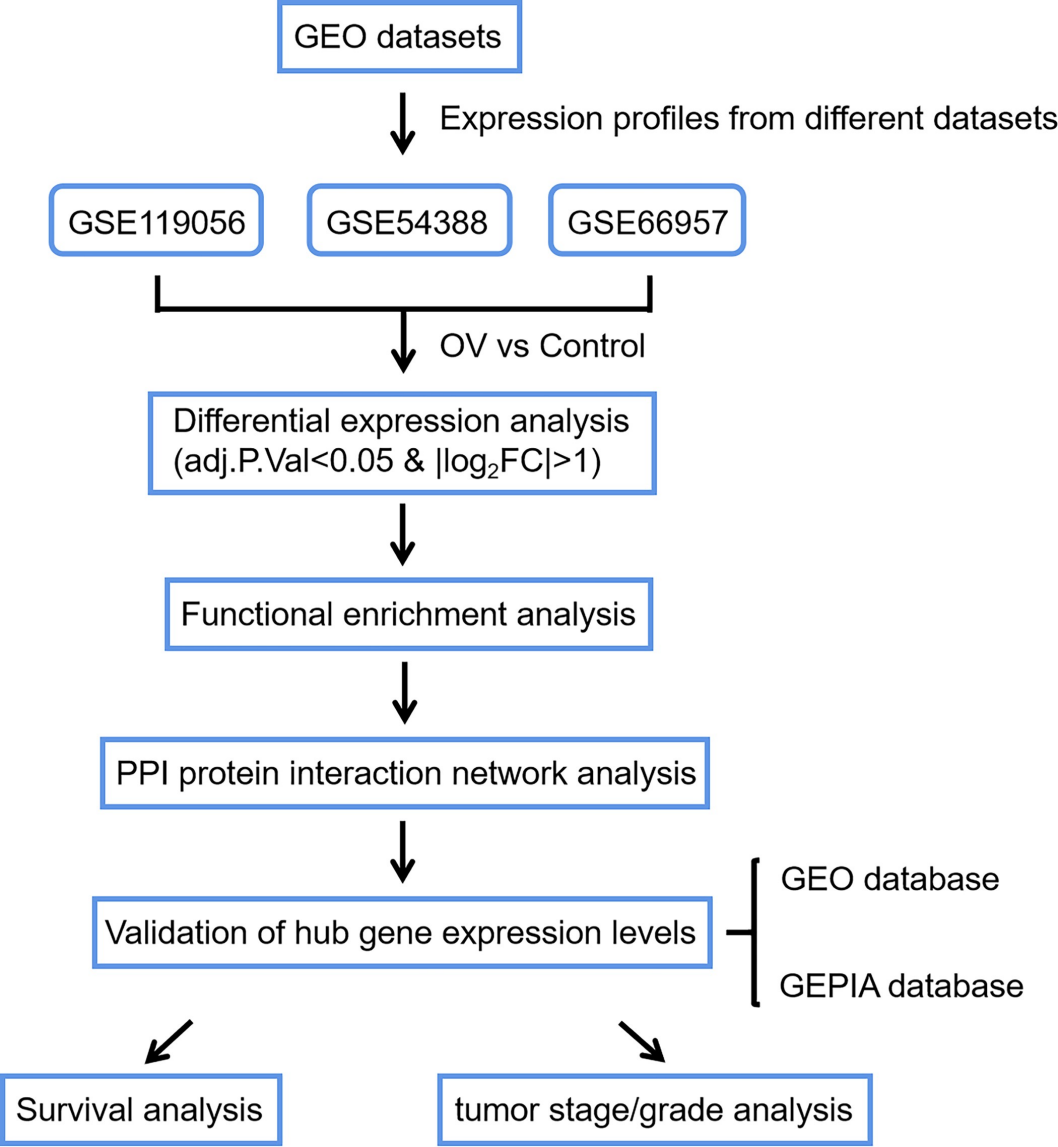
Flowchart illustrating the process of our research.

Overall, the process of our work was illustrated by a flowchart in Fig 8.

## Discussion

Despite significant advances in EOC treatment, including surgery, chemotherapy, radiotherapy, and novel targeted agents, EOC had remained an intractable cancer over the past several decades. Therefore, uncovering the etiological and molecular mechanisms underlying EOC is of vital importance for cancer therapy and prevention. For many years, bioinformatics analysis has been playing crucial roles in cancer study, and it facilitates the understanding of carcinogenesis by integrating data at the genome level with systematic bioinformatics methods.

Emerging single-celled RNA sequencing technologies offer unprecedented opportunities to analyze the interactions between cancer cells and the associated microenvironment. Modeling and characterization strategies based on differential regulatory networks were used to quantify and determine key genes for cancer drug resistance and to identify prognostic and predictive characteristics of cancer patients [16,17]. In an attempt of using inherited germline variants to predict clinical outcomes of cancer patient population, researchers constructed predictive models based on exome sequencing data to predict the risk of cancer recurrence. Gene signatures derived from the genes containing functionally germline variants significantly distinguished recurred and non-recurred patients. Germline genomic information could be used for developing non-invasive genomic tests for predicting patients’ outcomes [18].

Among multiple bioinformatics strategies, microarray gene expression profiling has been widely applied to explore DEGs involved in tumorigenesis, diagnosis, and therapeutic approaches [19]. In this study, we first screened DEGs from three independent GEO datasets, and implemented GO-KEGG pathways enrichment analysis. A PPI network was constructed in the STRING database and the top 20 hub genes were selected in Cytoscape. We then implemented literature retrieval of the 20 genes in Pubmed. Five genes were found having only one or two research papers published previously, including CDC45, CDCA5, KIF4A, ESPL1, and SPAG5. Although there was only one research paper found focusing on the gene SPAG5, the correlation of this gene with the ovarian cancer had been explored deeply in the paper. Therefore, we focused on the other four hub genes in our subsequent research. The relative expression of the four genes, CDC45, CDCA5, KIF4A, and ESPL1, was detected in GEO and GEPIA databases, results indicating that all the four hub genes were up-regulated in EOC tissues with statistical significance. Clinical stage analysis indicated that the expression of these four genes decreased gradually with the continuous progression of EOC. Survival curves illustrated that patients with a lower level of CDCA5 and ESPL1 had better overall survival and progression-free survival compared to patients with higher expression. Therefore, these two hub genes, CDCA5 and ESPL1, could be utilized as potential diagnostic indicators for EOC.

Cell-division cycle-associated 5 (CDCA5), also known as sororin, is thought to play a critical role in ensuring the accurate separation of sister chromatids during the S and G2/M phases of the cell cycle through interactions with cohesin and cdk1 [20,21]. CDCA5 has also been shown to interact with ERK as well as cyclin E1, a critical regulator of the G1/Smitotic checkpoint [20–22]. Recent studies have correlated the expression of CDCA5 with tumorigenesis and tissue invasion in several cancers.

Regarding lung cancer, several researches confirmed that CDCA5, exhibiting high specificity and sensitivity to distinguish malignant lesions from non-malignant tissues and associated with poor survival, could be identified as predictive biomarkers for tumorigenesis and poor prognosis of lung adenocarcinomas [23,24]. In study performed by Nguyen et al, suppression of CDCA5 expression inhibited the growth of lung cancer cells; concordantly, induction of exogenous expression of CDCA5 conferred growth-promoting activity in mammalian cells. Their data suggested that transactivation of CDCA5 and its phosphorylation at Ser209 by ERK played an important role in lung cancer proliferation, and that the selective suppression of the ERK-CDCA5 pathway could be a promising strategy for cancer therapy [22].

In researches of hepatocellular carcinoma (HCC), CDCA5 was also found to be up-regulated in HCC cells, and related to poor prognosis [25]. CDCA5 participated the promotion of HCC cells proliferation, migration, and invasion, palying a tumor-promotive role and being a potential therapeutic target for patients with HCC [26,27]. Besides, CDCA5 was found to be transcribed by E2F1, and could promote oncogenesis by enhancing cell proliferation and inhibiting apoptosis via the AKT pathway in HCC [28]. Another research found that increased CDCA5 expression was associated with increased tumor diameter and microvascular invasion in HCC [29]. Furthermore, silencing of CDCA5 inhibited cell proliferation and induced G2/M cycle arrest *in vitro*, and CDCA5 down-regulation in xenograft model impeded HCC growth *in vivo*. CDCA5 depletion decreased the levels of ERK 1/2 and AKT phosphorylation *in vitro* and *in vivo*. Taken together, theses results indicated that CDCA5 might act as a novel prognostic biomarker and therapeutic target for HCC [30].

In addition, it has also been confirmed that CDCA5 was significantly upregulated in breast cancer, bladder cancer, oral squamous cell cancer, urinary tract carcinoma, head and neck squamous cell carcinoma, and esophageal squamous cell carcinoma, and the high expression of CDCA5 was closely related to pathological stages and poor prognosis of patients [31–36].

ESPL1, also known as extra spindle poles-like 1 protein or separin, plays a central role in chromosome segregation by cleaving the cohesin complex at the onset of anaphase [37], and altered ESPL1 activity is correlated with aneuploidy and cancer [38]. At present, the results on the roles of ESPL1 in cancers are conflicting.

ESPL1 expression has been found to be upregulated in a wide range of cancers and high expression of ESPL1 is associated with a loss of key tumor suppressor gene P53, which further contributes to the progression of mammary adenocarcinomas [39,40]. The research conducted by Finetti et al reinforced that ESPL1 was a candidate oncogene in luminal B breast cancer, and the expression of ESPL1 might represent a promising therapeutic approach for the poor-prognosis tumors [41]. Genomic analysis of transitional cell carcinoma (TCC) by both whole-genome and whole-exome sequencing of 99 individuals with TCC found frequent alterations in ESPL1 [42]. Chen et al found that ESPL1 may be associated with bladder cancer development and recurrence [43]. In addition, Liu et al. identified 7 pivotal genes involved in endometrial cancer prognosis and constructed a prognostic gene signature, among which ESPL1 was one of the genes that were viewed as risky prognostic genes [44]. ESPL1 expression was found to be increased in endometrial cancer (EC) tissues, but the clinical significance and functional mechanism of ESPL1 in EC remains to be verified [44]. Nevertheless, it has also been reported that ESPL1 plays an opposite role in gastric adenocarcinoma. ESPL1 expression was negatively correlated with gastric adenocarcinoma pathologic stage progression, and the high expression of ESPL1 was significantly correlated with favorable outcomes [45]. Further work is required to resolve the conflicting roles of ESPL1 in cancer and determine its functions in cancers including the ovarian cancer.

In our research, among the selected 4 hub genes up-regulated in EOC, CDC45L and KLF4A had no significant correlations with OS and PFS, indicating that there were great gap between DEGs and functional genes.

The CDC45 gene, also known as CDC45L, exerts an important role in DNA replication including initiation and elongation phases as well as late G1 phase [46,47]. Defect in replication functions leads to DNA damage and chromesome rearrangement [48,49]. In most studies, CDC45 was reported over-expression and enrichment in pathways such as cell cycle arrest in several cancers [50–53]. However, the correlation of CDC45 expression with PFS and OS of cancer patients was different at different tumor stage. The expression of CDC45 was higher in colorectal cancer tissues than adjacent mucosa tissues, but colorectal patients with CDC45 low expression in tumor samples had worse relapse-free survival and overall survival, indicating that CDC45 might act as oncogene in early stage but have suppressor effects on cancer in advanced stage [54]. In our study, CDC45 expression was higher in EOC samples, but showed no statistical influence on PFS and OS. Previous researches and out study indicate that the role of CDC45 involved in cancer progression required further studies to confirm.

Regarding KIF4, it plays important roles in DNA repair and DNA replication maintaining genetic stability and is also essential for regulation of mitosis and meiosis [55–57]. Abnormalities in KIF4 are associated with a variety of diseases, including cancer, HIV infection, Alzheimer’s disease [58]. Interestingly, KIF4 is abnormally expressed in various cancers, where KIF4 is often up-regulated but can also be down-regulated in certain cancers, suggesting distinctive regulatory mechanisms for different cancers. The expression of KIF4 was significantly up-regulated in hepatocellular carcinoma, cervical cancer, lung cancer, pancreatic carcinoma, and oral squamous cell carcinoma [59–63]. In other types of cancer, KIF4 has opposite effects, with decreased expression in gastric cancer, multiple myeloma, acute myeloid leukemia, and osteosarcoma [64–66]. In our research, KIF4 was found over-expressed in EOC tissues, but no statistical significance was observed in correlation with survival. Therefore, KIF4, biologically relevant to oncogenic processes, has different prognostic implications for the survival of various solid tumors. Additionally, the underlying regulative mechanisms remain as open questions. At present, the research of KIF4 in cancer has been focused on expression testing, and the specific mechanisms of action are still poorly understood. Further mechanistic studies are needed to support the use of KIF4 as a therapeutic target.

In summary, our study identified 4 novel hub genes (CDCA5, ESPLA, CAC45, and KLF4A) by using integrative analysis of gene expression profiling in EOC based on GEO database. Further survival curves illustrated that higher expression of CDCA5 and ESPL1 predicted poorer overall survival and progression-free survival in EOC, while CDC45 and KLF4A had no significant correlations. Literature retrieval showed that the expression of CDCA5 was associated with tumorigenesis and tissue invasion in a variety of cancers, while the results on the roles of ESPL1, CDC45, and ESPL1 in cancers were conflicting. CDCA5 and ESPL1 may act as biomarkers and potential therapy targets in EOC.

Our study provided two potential targets for future experimental and clinical investigation of the development and progression of ovarian cancer. Further studies are merited to explore the biological functions of these two genes and to clarify the underlying molecular mechanisms involved in the pathogenesis of EOC. This would be of great help to early diagnosis and targeted therapy of ovarian cancer. In the future, we could further analyze the ceRNAs for the two potential targets genes. Construction of the ceRNA network may help elucidate the regulatory mechanisms underlying the pathogenesis of ovarian cancer. Candidate lncRNAs, miRNAs, and mRNAs participating in the ceRNA network could be further evaluated as potential therapeutic targets and prognostic biomarkers for ovarian cancer [67–69].

There are several limitations in our study as follows. First, there is an urgent need for biological experiments to validate our results because our research is based on data analysis. We are currently collecting tissue samples from EOC patients in China to verify our current analysis. Sample collection and validation have been delayed due to objective reasons such as COVID-19. Second, we lack the molecular mechanisms for these genes, and we will incorporate these for further exploration.

## Conclusions

In conclusion, our study provided a comprehensive bioinformatics analysis of DEGs, which may have the potential to serve as reliable molecular biomarkers for the diagnosis and prognosis of EOC. Two genes, CDCA5 and ESPL1, were validated to be up-regulated in EOC samples, and high expression of these two genes were related with poor overall survival and progression-free survival. CDCA5 and ESPL1 may act as biomarkers and potential therapy targets in EOC. Further studies are merited to explore the biological functions of these two genes and to clarify the underlying molecular mechanisms involved in the pathogenesis of EOC.
